# CD133 Act as an Essential Marker in Ovarian Carcinogenesis

**DOI:** 10.31557/APJCP.2021.22.11.3525

**Published:** 2021-11

**Authors:** Dzul Ikram, Rina Masadah, Berti J Nelwan, Andi Alfian Zainuddin, Mahmud Ghaznawie, Syarifuddin Wahid

**Affiliations:** 1 *Department of Anatomical Pathology, Faculty of Medicine, Universitas Hasanuddin, Makassar, Indonesia. *; 2 *Department of Histology, Faculty of Medicine, Universitas Muslim Indonesia, Makassar, Indonesia. *; 3 *Department of Public Health, Faculty of Medicine, Universitas Hasanuddin, Makassar, Indonesia. *; 4 *Department of Anatomical Pathology, Faculty of Medicine, Universitas Muhammadiyah Makassar, Makassar, Indonesia. *; 5 *Department of Anatomical Pathology, Faculty of Medicine, Universitas Muslim Indonesia, Makassar, Indonesia. *

**Keywords:** CD133, cancer stem cells, carcinogenesis, ovarian cancer

## Abstract

**Objective:**

To analyze the role of cancer stem cells (CSC) in ovarian carcinogenesis through the identification of CD133 expression in the normal ovary (NO), serous cystadenoma (SC), borderline serous tumour (BST), low-grade serous carcinoma (LGSC), and high-grade serous carcinoma (HGSC).

**Materials and methods:**

A total of 48 tissue samples contain 5 NO, 10 SC, 5 BST, 8 LGSC, and 20 HGSC were stained with anti-CD133 antibody by immunohistochemical protocol. The difference in the H-score of CD133 expression between groups and their relationship to age, histomorphology, and localization was analyzed.

**Results:**

CD133 expression varied among tumor groups, with clinicopathologic parameters showing diverse associations (age p = 0.773; histomorphology p = 0.001; and localization p = 0.026). The comparison of CD133 H-scores differed significantly between each group (p = 0.0031), in which precursor and malignant lesions possessed more robust CD133 expression.

**Conclusion:**

The presence of CD133 cellular expression and localization in different types of serous ovarian tumours suggests that these markers are involved in ovarian tumorigenesis.

## Introduction

Ovarian cancer (OC), with an estimated 295,000 cases and 184,000 deaths worldwide, is the 8th most prevalent cancer diagnosis and lethal gynaecological malignancy affecting a large female population (Bray et al., 2018). In Asian countries, 110,526 cases have typically been registered in the standardized OC incidence rate. Indonesia was the third-highest in OC deaths after China and India (Razi et al., 2016). Approximately 60 per cent of the women with OC already have an advanced stage of the disease at the time of presentation, and in such cases, the 5-year survival rate is just 29 per cent.

The cause for OC is still not entirely understood, but many factors that play a role in its development have been identified (Beral et al., 2008). Age, lack of birth, and family history serve as risk factors, while protective factors include parity, oral contraceptives, lactation, late menarche, and early menopause. However, direct causality is still not proven between them and OC (Tsilidis et al., 2011). Various factors, including atypical clinical symptoms, insufficient early detection modalities, late-onset diagnosis, chemoresistance, recurrence, and metastatic spread, lead to high mortality of ovarian cancer (Burges and Schmalfeldt, 2011; Chen et al., 2017; Klapdor et al., 2017).

Approximately ninety per cent of OC are of epithelial origin. Based on the histological and molecular findings, OC was classified into type 1 and type 2. Type 1 is slow-growing, low grade, and genetically stable, while type 2 is more aggressive and variably alters the p53 molecular levels (Malpica and Wong, 2016). According to the current WHO classification, serous tumours are the most common histologic type of female genital tract tumours (Kurman et al., 2014). Low-grade serous carcinoma (LGSC) grows indolently from precursor lesions: serous cystadenoma (SC) to a borderline serous tumour (BST) (Hatano et al., 2019). The micropapillary pattern with or without invasion is a histopathological hallmark used to differentiate between BST and LGSC (Kurman et al., 2014). In comparison, high-grade serous carcinoma (HGSC) has been demonstrated to derive from a stepwise progression of intraepithelial tumour serous tubal lesions (STIC) (Lee et al., 2007). KRAS-BRAF and TP53 mutations are involved in these two entities, respectively (Tsuchida et al., 2016; Hatano et al., 2019). Their characterization is based on a histologic analysis showing that tumour cells resemble a sort of epithelial-like cells that most likely come from the fallopian tube epithelial (FTE) or ovarian surface epithelial (OSE) (Kurman et al., 2014). Both cells express cancer stem cells (CSC) markers, including ALDH1, LGR5, LEF1, CK6B, and CD133 (Auersperg, 2013). 

CD133 or Prominin-1 is a membrane-anchored and cell surface protein first discovered in mouse and human stem cells (Shmelkov et al., 2005), which indicates the presence of cancer stem cells in tumorous tissue (Barzegar et al., 2019). It is often challenging to predict the likelihood of malignant transformation based on histopathological findings alone. Therefore, it is essential to include an additional measure to assess potential malignant transformation in an ovarian precursor lesion. The identification of CD133+ cells provides insight into tumour biology related to metabolism, apoptosis, autophagy, tumorigenesis, metastases, and chemoresistance (Li, 2013). It is also expected to have practical implications for assessing the risk of malignancy in precursor lesions. Thus, this study aimed to analyze the role of CSC in ovarian carcinogenesis through CD133 expression in normal ovaries, precursor lesions, and malignant lesions.

## Materials

The research design was an observational study with a cross-sectional approach through histomorphological and immunohistochemical analysis of precursors and malignant lesions. We analyzed 48 samples consisted of 5 normal ovaries, ten SC, five BST, eight LGSC, and 20 HGSC. All samples were embedded paraffin and obtained from patients in three anatomical pathology centres in Makassar (Wahidin Sudirohusodo Hospital, Hasanuddin University Hospital, and Makassar Pathology Diagnostic Center). Samples were collected from January 2017 to December 2020. The use of stored biological material has been approved by The Research Ethics Committee of the Faculty of Medicine Universitas Hasanuddin.

*Histomorphological analysis*

Specimens were first fixed in 10% buffered formalin, then embedded in paraffin, sectioned at 5 µm, stained with hematoxylin-eosin, and placed on glass slides for histopathological study. Samples have been classified according to the WHO Classification of Female Genital Tumours 5th edition.

*Immunohistochemical procedure*

Tissue samples at 3 µm were sectioned from paraffin-embedded material. The entire slides were treated according to standard protocols, and serial cuts were used for immunohistochemical reactions (IHC). After deparaffinization in xylene and rehydration in various ethanol concentrations, slides were placed in 3% hydrogen peroxide for 3 minutes then washed in tap water. Heat-induced epitope retrieval was then performed by putting samples into the microwave for 10 minutes. Specimens were then allowed at room temperature and washed with Tris Buffer Saline and 1% protein blocking solution for 10 minutes. All specimens were then incubated in polyclonal rabbit anti-human CD133 antibody (1:200) for 90 minutes at room temperature. The samples were then incubated in secondary antibody (Ultratek Complete HRP Anti-Polyvalent – LSAB) for ten minutes. Next, all samples were then incubated with chromogen Diaminobenzidine (DAB) and washed in running water for 5 minutes, then immersed in a solution of Hematoxylin Modified Lillie’s Mayer as counterstaining. Finally, all slides were then covered on a deck glass and further analyzed under the Microscope Binocular Olympus CX-43. Each reaction was accompanied by positive control (pancreas). Negative control (absence of primary antibody) was used in conjunction with the incubation of the samples.

*Quantitative Analysis*

Two gynaecological pathologists analyzed CD133 expression without knowing the clinical data of each other. Assessment based on the percentage of stained cells (extensity) in 10 ‘hotspot’ high power field (0-100), intensity (0-3), and histochemical score (H-Score) (0-300) by multiplying extensity and intensity. CD133 expression is categorized as a low or high expression based on the median H-score determined as a cut-off value (Keymoosi et al., 2014). Positive expression of CD133 will appear brown stained on the membrane and cytoplasm of tumour cells. The percentage of stained cells was divided into 3 levels: 1 (0% CD133 + cells), 2 (<10% CD133 + cells), and 3 (> 10% CD133 + cells) (Cioffi et al., 2015). The CD133 staining intensity levels were classified into four categories: 0 (no stained/negative cells), +1 (weak staining or only visible with 40x objective lens magnification), +2 (moderate or visible staining with 10x objective lens magnification), + 3 (strong or visible stain with 10x objective lens magnification) (Mardani et al., 2020). All slides were observed at x400 magnification using a light microscope (Olympus CX-43).

*Statistical Analysis*

All data were analyzed using GraphPad Prism software version 6. Values are expressed in mean + SD. The Kruskal-Wallis test, followed by the Mann-Whitney test, was used to assess the mean H-score difference between the test groups. A p-value <0.05 was considered statistically significant.

## Results

*Sample Characteristics*

Samples were obtained from 48 cases consisting of 5 normal ovaries, 10 SC, 5 BST, 8 LGSC, and 20 HGSC. The mean age in the SC and BST premalignant lesion groups were 42.2 + 8.63 SD and 41.6 + 15.8 SD, ranging between 24-56 years and 26-66 years, respectively. Meanwhile, the age range of LGSC and HGSC malignant lesions was between 23-58 years and 24-77 years with mean age 42 + 13.4 SD and 50.4 + 12.9 SD. As shown in table 1, there was no significant age difference between the CD133-low expression tumour group and the CD133-high expression p 0.773 group.

Histomorphological and localization analysis of the expression status on CD133-stained cells can be seen in Table 1. There was a significant difference between histomorphology and CD133 expression status (p=0.001). We then assessed the differences in the location of expression in each tumour group. The data showed significant differences in the site of CD133 expression based on the type of histomorphology with a p-value=0.026. The data indicates a relationship between histomorphology and localization.

*Immunohistochemical profiles of CD133 in ovarian tumours*

**Figure 1:**
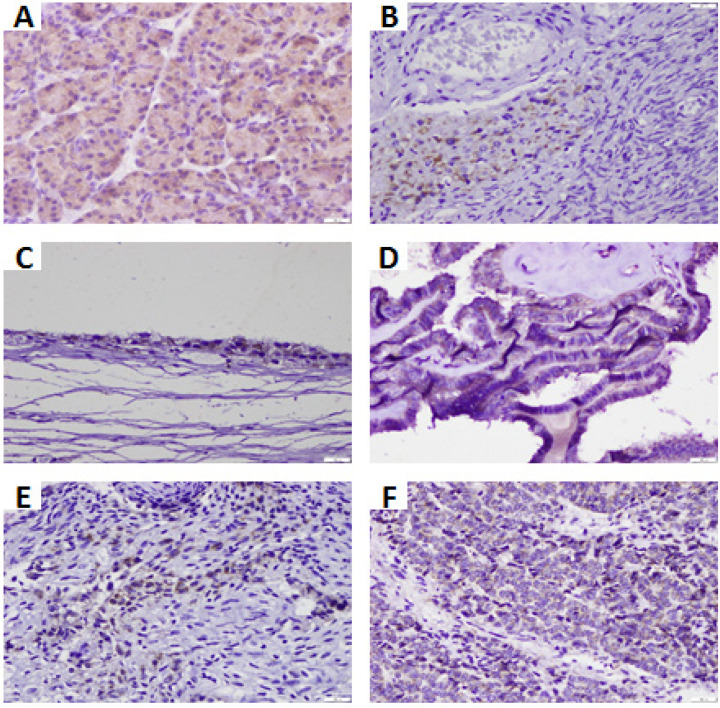
Representative Samples of CD133+ Cells among Tumour Group. (A) Positive control in human pancreas tissue; (B) Normal ovary showed positive expression located in stromal cells with apical/membranous pattern; C) Serous Cystadenoma expressed CD133 in epithelial lining cells with membranous pattern; (D) Borderline Serous Tumor showed positive CD133 expression diffuse strong cytoplasmic pattern; (E) Low-grade Serous Carcinoma; and (F) High-grade serous carcinoma expressed CD133+ve in tumour cells, respectively. Note that staining intensity and extensity in HGSC are greater than LGSC. Magnification 400x; Scale bar 100 µm

We identified CD133+ cells in normal ovaries, benign and precursor lesions, and malignant lesions (Figure 1A-1F). It appeared that the intensity and the percentage of the area of CD133 stained varied in each group. The most robust expression was observed in BST, HSGC, and LGSC, located in the cytoplasm. The SC lesion shows weak CD133 expression on the membrane or apical tumour cells. The expression of CD133 in normal ovaries showed a membrane staining pattern in the stromal area with weak intensity. Furthermore, we analyzed the CD133 positive rate values in each histomorphological group (Table 1). Positive rates> 80% were indicated by the SC and BST precursor lesion groups and the LGSC and HGSC malignant lesion groups, where BST and HGSC showed the highest values.

**Figure 2:**
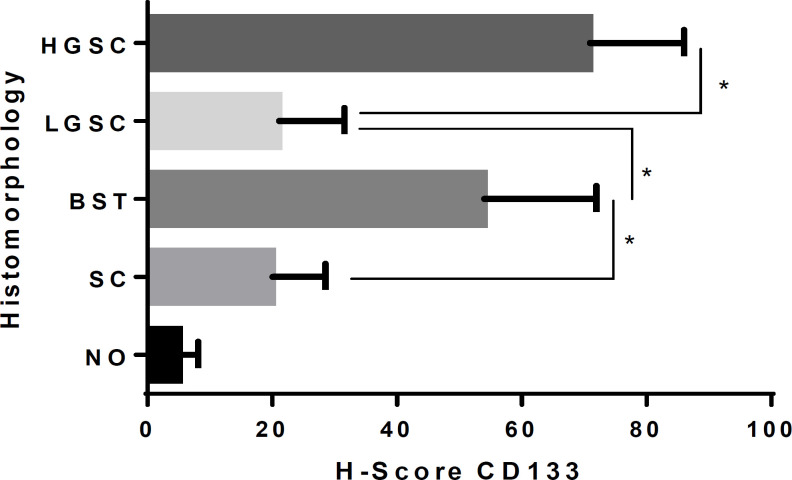
CD133 H-score Comparison Regarding Histomorphology. There was a statistically significant difference among the groups of tumours (Kruskal-Wallis test p 0,0031). *Post-Hoc analysis Mann-Whitney showed a significant difference between SC vs. BST p=0.0366, BST vs. LGSC p=0.0451, and LGSC vs. HGSC p=0.0221

We compared H-scores for CD133 expression between the normal ovaries, benign, precursor, and malignant lesions. The results showed that there was a statistically significant difference between each tumour group p 0.0031. Subsequent Mann-Whitney analysis revealed significant H-score differences between the three groups, namely SC vs. BST p 0,0366, BST vs. LGSC p 0,0451, and LGSC vs. HGSC p 0,0221, while NO vs. SC not statistically significant p 0,1522 (Figure 2).

**Table 1:** Association of CD133 Expression and Characteristics of Samples

Characteristics of Sample	n (%)	CD133 Expression		p
		Low n (%)	High n (%)	
Age (years)				
Mean+SD	47.23 (12.9)	47.71 (13.6)	46.75 (12.5)	0.802^a^
<47	25 (52.1)	13 (54.2)	12 (50)	0.773^b^
>47	23 (47.9)	11 (45.8)	12 (50)	
Histo-Morphology				
Normal ovary	5 (10.4)	5 (20.8)	0 (0)	0.001^c^
Serous cystadenoma	10 (20.8)	7 (29.2)	3 (12.5)	
Borderline serous cystadenoma	5 (10.4)	0 (0)	5 (20.8)	
Low grade serous carcinoma	8 (16.7)	6 (25)	2 (8.3)	
High grade serous carcinoma	20 (41.7)	6 (25)	14 (58.3)	
Localization				0.026^c^
Apical or membranous	13 (27.1)	8 (33.3)	5 (20.8)	
Cytoplasmic	29 (60.4)	10 (41.7)	19 (79.2)	
NA*	6 (12.5)			
Total	48 (100)	24 (50)	24 (50)	-

*NA, Not Assessable; ^a^, Unpaired T-Test; ^b^, Chi-square; ^c^, Fisher’s Exact Test

**Table 2:** The Distribution of CD133 Positive Rates among Tumour Groups

Group of Tumours	N	CD133 Expression		Positive Rates (%)
		Negative	Positive	
Normal Ovary	5	2	3	60
Serous Cystadenoma	10	2	8	80
Borderline Serous Tumour	5	0	5	100
Low-Grade Serous Carcinoma	8	1	7	87.5
High-Grade Serous Carcinoma	20	1	19	95
Total	48	6	42	87.5

## Discussion

This study presented a relatively younger mean age in the premalignant lesion group than the malignant lesion group. (Longacre et al., 2005) noted that the mean age of patients with serous tumours of low malignant potential was 42.3 years. In malignant, Arora et al., (2018) reported age at diagnosis to be 50-79 years, whereas our findings showed the mean age in the HGSC group falls within this range. These indicate the age trend of patients with precursor lesions and malignant ovarian epithelial lesions in the 4th to 5^th^ decade of life. Although the relationship between age and outcome of ovarian cancer is unclear, several studies have reported that younger age showed better outcomes than older age due to aggressive treatment (Ries, 1993; Arora et al., 2018). Other risk factors that also play a role include menstrual aspects, endometriosis, family history, and BRCA mutations, while parity, contraceptive methods, and lactation are protective factors (Momenimovahed et al., 2019).

Our evaluation of the staining pattern with CD133 expression in the various ovarian tumour groups showed a statistically significant difference (see Table 1). The apical or membranous staining pattern was predominantly found in normal ovarian located in the stromal spindle cells and SC tumour cells. In contrast, malignant lesions, especially HGSC, showed diffuse staining patterns in the cytoplasm. In line with these findings, (Huang et al., 2015) reported CD133 expression in the cytoplasm and nucleus of tumour cells was associated with a poor prognosis in non-small cell lung cancer. Ferrandina et al., (2009) also noted a tendency for ovarian cancer with a CD133 diffuse cytoplasmic staining pattern to have a worse prognosis than with the apical cytoplasmic pattern. CD133 is a transmembrane protein in non-tumorous cells, while in tumorous lesions, it can be internalized into the cytoplasm and nucleus through the endocytosis pathway (Planque, 2006). CD133 is thought to play a role in regulating transcription factors in DNA through complex interactions with molecular pathways that control tumor cells’ proliferation and differentiation (Huang et al., 2015). However, the exact mechanism remains unclear and needs further exploration.

CSCs, also known as tumour-initiating cells, are a small number of cells capable of self-renewal and differentiation to form new cell populations (Gupta et al., 2009). OC tumorigenesis is quite complicated, usually involving precursor lesions. OC can also apply various stem cell proteins in complex interactions (Suster and Virant-Klun, 2019). The presence of CSCs in ovarian cancer is vital in triggering tumour development towards malignancy and disseminating tumour cells (Amaya Padilla et al., 2019). In this study, we analyzed the presence and role of CD133, as a marker of CSC, in ovarian cancer tumorigenesis using the IHC method. Here we analyzed CD133 expression and its positive rates in normal ovaries, precursor lesions, and malignant lesions, as shown in Figure 1 and Table 2. 

Expression of CD133 on normal ovarian and benign SC lesions showed the distribution in the stromal-spindle cells and OSE-epithelial lining area, respectively. This data is consistent with the research (Zhang et al., 2012), which also identified CD133 expression in OSE and normal ovarian stroma. Various studies have detected tiny embryonic-like stem cells in normal ovaries of adult women in the OSE area (Parte et al., 2011; Virant-klun et al., 2013). An attractive hypothesis is that CSC can undergo a phenotypical transformation from epithelial to mesenchymal-like cells (Suster and Virant-Klun, 2019). In BST precursor lesions, malignant LGSC and HGSC lesions showed strong CD133 expression in the cytoplasm even down to the tumour cell nucleus. (Hashimoto and Aoyagi, 2014) disclosed that cytoplasmic CD133 expression in gastric cancer is related to tumour progression and metastasis. These findings indicate the process of internalizing some CD133 proteins from the cell membrane to the cytoplasm and nucleus. CD133 signaling in the cytoplasm regarding cellular starvation or stress likely enhances tumour cell survival (Jang et al., 2017). Although the exact mechanism is not clear, several reports discuss the possible mechanisms associated with internalization of proteins from the membrane to the cytoplasm and nucleus, namely (i) endosome mediation, (ii) mediation of chaperone-like proteins, (iii) mediation of γ-secretase enzymes (Wells and Marti, 2002; Bryant and Stow, 2005).

Our findings designated that there was a positive rate variation in each tumour group. The higher positive rate of CD133 was mainly expressed in precursor and malignant lesions over normal and benign ones (Table 2). We assume that the extent of malignancy impacts the status of CD133 expression in terms of positivity, intensity, and stained tumor cells’ extensity. Although contradictory evidence is still available, there is a link between CD133 expression status in ovarian cancer and several other clinicopathological factors. A recently published study by Pelupessy et al., (2019) showed that CD133-negative expression in ovarian cancer correlates with a higher chemoresistance score and has a better ROC curve. In comparison, increased CD133 expression was observed during OCSC induction and chemotherapy drug treatment (Liu et al., 2020). Consistent with the latter, (Yifeng et al., 2018) used meta-analysis to provide more significant evidence and concluded that CD133 expression along with CD44 is indicative of advanced FIGO level, degree of differentiation, and chemotherapy resistance in ovarian cancer cells.

We further analyzed the histomorphological association of ovarian tumours with H-score CD133 expression, which showed a statistically significant difference (see Figure 2). The BST group showed the highest CD133 H-score compared to the other groups. Mann-Whitney analysis showed significant differences between SC vs. BST, BST vs. LGSC, and LGSC vs. HGSC. Our findings indicated an increase in the H-score of CD133 and a shift in the ovarian tumor’s malignancy status. In typical ovaries or benign tumours, the levels of CD133-1 and CD133-2-expressing cells were substantially lower than those of ovary carcinomas (Ferrandina et al., 2008). Following the previous data, (Dewayani et al., 2020) also noted that BST with FIGO stage more than IA expressed CD133 higher than BST with IA. Furthermore, evidence of an association of CD133 expression with ovarian cancer morphology was reported by Zhang et al., (2012), in which HGSC entities expressed this protein higher than LGSC and other malignant ovarian tumors of epithelial origin.

Due to ovarian cancer heterogeneity, utilizing only a single CSC IHC marker would restrict the authors from interpreting complex relationships and ovarian carcinogenesis interactions. Another drawback of this study is the limited sample size, which restricts the analysis scope to larger and more homogeneous groups that we believe can impact the accuracy of existing data.

On this basis, we conclude that the existence of CD133 cellular expression and localization in various groups of ovarian tumours of serous epithelial origin, ranging from benign, borderline, and malignant entities, indicates the involvement of these markers in ovarian cancer tumorigenesis. However, this study has limitations because it only uses a single CSC marker. Histopathologic determination of tumour grading still has an aspect of subjectivity. The CD133 immunohistochemical modality, together with histopathological findings on premalignant precursor lesions, can better identify and assess malignancy risk.

## Author Contribution Statement

DI, RM, BJN contributed to the conceptualization and design of the method; DI, RM, BJN, AAZ engaged with the curation of data, analysis, and interpretation of the findings; MG and SW reviewed it extensively for intellectual content and editing; All authors reviewed and approved the final version of the article. 

## Funding Statement

The study was supported by the Faculty of Medicine, Hasanuddin University, Makassar, Indonesia, through an internal research grant 2020.

## Study Approval

This manuscript is a part of an approved DI’s thesis on the academic program for anatomic pathology specialist.

## Availability of Data

The datasets used in this study are available upon reasonable request from the corresponding author. 

## Ethical approval

The Ethics Committee of the Faculty of Medicine, University of Hasanuddin, Makassar, Indonesia permitted this study (Registry No. 309/UN4.6.4.5.31/PP36/2021) with a waiver of informed consent.

## Conflict of Interest

All authors state that they have no conflicting interests.
